# Plasmonic Gold Nanorods Coverage Influence on Enhancement of the Photoluminescence of Two-Dimensional MoS_2_ Monolayer

**DOI:** 10.1038/srep16374

**Published:** 2015-11-17

**Authors:** Kevin C. J. Lee, Yi-Huan Chen, Hsiang-Yu Lin, Chia-Chin Cheng, Pei-Ying Chen, Ting-Yi Wu, Min-Hsiung Shih, Kung-Hwa Wei, Lain-Jong Li, Chien-Wen Chang

**Affiliations:** 1 Research Center of Applied Sciences (RCAS), Academia Sinica, Taipei, 11529, Taiwan; 2 Department of Photonics, National Chiao Tung University (NCTU), Hsinchu, 30010, Taiwan; 3 Department of Materials Science & Engineering, National Chiao Tung University Hsinchu, 30010, Taiwan; 4 Physical Science and Engineering Division, King Abdullah University of Science and Technology (KAUST), Thuwal, 23955-6900, Kingdom of Saudi Arabia; 5 Department of Biomedical Engineering and Environmental Sciences, National Tsing Hua University (NTHU), Hsinchu, 30013, Taiwan; 6 Department of Photonics, National Sun Yat-sen University, Kaohsiung, 804, Taiwan

## Abstract

The 2-D transition metal dichalcogenide (TMD) semiconductors, has received great attention due to its excellent optical and electronic properties and potential applications in field-effect transistors, light emitting and sensing devices. Recently surface plasmon enhanced photoluminescence (PL) of the weak 2-D TMD atomic layers was developed to realize the potential optoelectronic devices. However, we noticed that the enhancement would not increase monotonically with increasing of metal plasmonic objects and the emission drop after the certain coverage. This study presents the optimized PL enhancement of a monolayer MoS_2_ in the presence of gold (Au) nanorods. A localized surface plasmon wave of Au nanorods that generated around the monolayer MoS_2_ can provide resonance wavelength overlapping with that of the MoS_2_ gain spectrum. These spatial and spectral overlapping between the localized surface plasmon polariton waves and that from MoS_2_ emission drastically enhanced the light emission from the MoS_2_ monolayer. We gave a simple model and physical interpretations to explain the phenomena. The plasmonic Au nanostructures approach provides a valuable avenue to enhancing the emitting efficiency of the 2-D nano-materials and their devices for the future optoelectronic devices and systems.

Two-dimensional materials have received considerable attention, mainly because of their unusual physical properties compared with their 3-D bulk forms. Graphene, the most famous member of the 2-D material family, exhibits excellent optical, electronic and mechanical properties such as transparency, conductivity, thermal dissipation and elasticity, and has been used in various applications^1^,^2^,^3^,^4^,^5^,^6^,^7^,^8^,^9^,^10^,^11^,^12^. However, the lack of a band gap in its pristine form has prompted a broad research for other 2-D semiconductor materials^13^. The 2-D transition metal dichalcogenide (TMD) semiconductor with electronic properties and a potential range of applications complementary to those of graphene, has recently attracted considerable attention^14^. Recently surface plasmon enhanced luminescence of the weak 2-D TMD atomic layers was investigated and reported to realize the next generation ulra-thin, flexible photonic and electronic devices^15^,^16^,^17^,^18^. However, the enhancement would not increase monotonically with increasing of plasmonic objects such as metal nanoparticles or resonators. Because overlapping between nanorods’ effective enhanced area and absorption of gold nanorods, the emission from the 2-D monolayers would decrease once the density of nanorods (or the number of nanorods within the pumping area) reaches the optimum value. In this study, we investigated metal coverage influence on PL of 2-D TMD monolayer, and achieve a high plasmon enhancement by optimizing the coverage of gold (Au) nanorods on the top of a molybdenum disulfide monolayer. The simple physical model was also illustrated to explain the behavior of plasmon enhancement in the 2-D TMD monolayer.

Molybdenum disulfide (MoS_2_) crystals that form hexagonal lattices are composed of vertically stacked weak van der Waals bonded S-Mo-S units. Because of low friction, the bulk form of MoS_2_ is widely used as a solid lubricant in the industry^19^. Recently the MoS_2_ crystal has been thinned to low-dimensional nanomaterials, and its novel physical phenomena and many potential applications were reported^13^,^14^,^18^,^20^,^21^,^22^,^23^,^24^,^25^,^26^,^27^,^28^,^29^,^30^,^31^,^32^,^33^,^34^. Since the distinctive photoluminescence (PL) was observed in 2-D MoS_2_ nanosheets, it became a potential gain material for the ultrathin, flexible, and transparent optoelectronic devices. The PL emission efficiency of the MoS_2_ gradually increases as the layer thickness decreases. However, one main bottleneck of low-dimensional MoS_2_ is their relatively lower light emission. The quantum yield of the monolayer MoS_2_ was reported to be approximately 4 × 10^−3^. Furthermore, the quantum yield of MoS_2_ is no more than the order of 10^−5^ when it possesses slightly over two layers, because of the behavior of their indirect band gap^25^. Hence, light enhancement in the monolayer MoS_2_ is critical for realizing the light emitters with the MoS_2_ gain material. Previous research has shown that the main peak intensity during tri-layer MoS_2_ PL spectra exhibits approximately two-fold enhancements through the application of a biaxial compressive strain^24^. And also, manipulating the dielectric environment of 2D-materials to enhance emission is proven to a viable method^35^. On the other hand, surface plasmonics owing to metal nanostructures had received huge attentions from researchers and scientists in various fields^36^,^37^,^38^,^39^,^40^,^41^,^42^,^43^,^44^,^45^,^46^,^47^,^48^,^49^. The surface plasmon wave, an electromagnetic wave generated in the interface between a dielectric medium and a metal layer upon irradiation, has the unique optical properties, because of the extremely strong light concentration in subwavelength metallic structures. The special optical waves had been applied to the advanced optical emitters, nanoscale optical antennas, ultra-compact plasmonic lasers, and optical trapping in a solar cell. Recent reports have shown numerous classic examples of surface plasmon enhanced PL in conventional luminescent materials such as ZnO^46^, GaN^47^, and InGaN^48^. In addition, recent studies utilizing silver plasmonic nanodisc arrays to enhance the emission of MoS_2_ showed that SPR enhancement is a promising way^16^. However, they did not discuss the density of metallic plasmonic structures to the enhancement of MoS_2_ emission. In the study, we demonstrated photoluminescence enhancement of monolayer MoS_2_ using plasmonic Gold (Au) nanorods. Figure 1 shows the schematic structure of a monolayer MoS_2_ and an Au nanorod. Through a careful selection of a suitable Au nanorod with a surface plasmon resonance (SPR) frequency matched the direct band gap of MoS_2_ (1.86 eV), the localized surface plasmon wave induced by the Au nanorod can spatially and spectrally be overlapped with the emission of a single-layer MoS_2_. This strong coupling leads to a large enhancement in the photoluminescence. As the density increases, we observed a decaying phenomenon of SPR enhancement. Therefore the nanorod density have to be optimized to achieve the high optical emission from the 2-D TMDC nanosheet. In the end, we will give a simple physical explanation to the decaying process.

## Results

**Figure 2:**
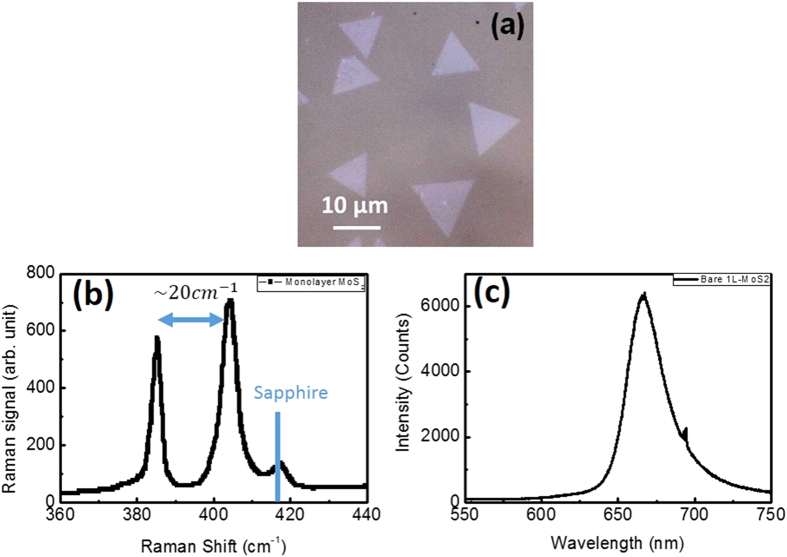
(**a**) Optical images of the MoS_2_ monolayer in the sapphire substrate. (**b**) Raman spectra for the MoS_2_ monolayer grown on sapphire substrates (excitation laser: 488 nm). (**c**) PL spectrum of monolayer MoS_2_. (excitation laser: 488 nm).

The growth of the monolayer MoS_2_ is based on the vapor phase reaction between MoO_3_ and S as reported by the previous studies^21^,^22^,^23^. Figure 2(a) shows the optical images of a monolayer MoS_2_ placed on the sapphire substrate. The size of the triangular MoS_2_ nanosheet is approximately 5 to 20 μm.

We investigated the optical properties of single-layered MoS_2_ by Raman and PL, where the measurements were performed in a Jobin-Yvon Horiba HR800 micro-Raman spectroscope at room temperature. Raman spectroscopy is a common tool for determining the precise number of layers in MoS_2_ sheets. Figure 2(b) showed that two distinguished Raman peaks, in-plane (E^1^_2g_ approximately 385 cm^−1^) and vertical-plane (A_1g_ approximately 405 cm^−1^) vibrations of Mo-S bonds in MoS_2_. The frequency difference Δ between these two modes was 20 cm^−1^. The photoluminescence of monolayer MoS_2_ was also measured with peak emission wavelength around 670 nm. Raman and PL signals of our MoS_2_ were both consistent with literature values.

To achieve the emission enhancement of the MoS_2_ nanosheets, we incorporated Au nanorods that have the local surface plasmon resonance (LSPR) around 670 nm wavelength into the devices. The Au nanorods were synthesized with excellent dispersibility in various organic solvents, including tetrahydrofuran and chloroform. With the nanorod preparation procedure, the gold nanorods can be coated on the top surface of MoS_2_ nanosheets without aggregation, and prevent the decoration effect from the small gold nanoparticles on the MoS_2_ monolayer^50^,^51^.

**Figure 3:**
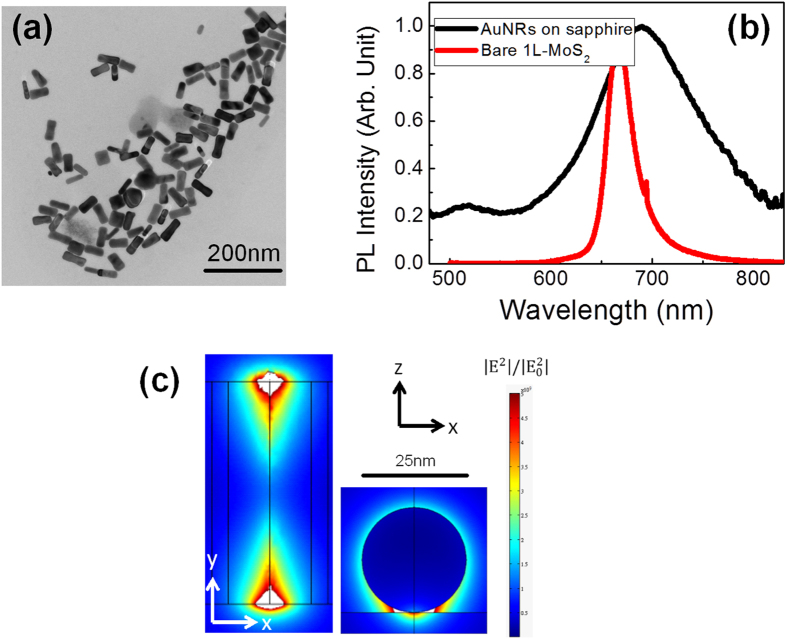
(**a**) TEM image illustrating the gold nanorods with a 57-nm length and 25-nm width on average. (**b**) The extinction spectra of gold nanorods on the sapphire substrate and the PL spectra of the MoS_2_ monolayer grown on the sapphire substrate. (**c**) Calculated near-field optical intensity map of a gold nanorod with a length of 57 nm and width of 25 nm laying on the sapphire substrate.

In the experiments, we used a toluene suspension of gold nanorods as an optical absorber, and the averaged size of these Au nanorods is 57 nm in length and 25 nm in diameter. Figure 3(a) shows the transmission electron microscopy (TEM) images of the high density Au nanorods in the solvant. To understand the optical absorption of the Au nanorods, the nanorods were spin coated on a double-polished sapphire substrate, and were then examined in a spectral range of 480–830 nm wavelength by using a tungsten–halogen source. The black curve in Fig. 3(b) shows the absorption spectrum of the Au nanorods measured by the monochromator. The broad longitudinal surface plasmon resonance of the nanorods mainly covers the wavelength from 600 to 800 nm. The red curve in Fig. 3(b) displays the PL spectrum of the monolayer MoS_2_ with an emission peak approximately 670 nm, which has an overlapping with the LSPR spectrum of the Au nanorods. When the energy transfers from the excitons in the MoS_2_ material to the metal nanorods through localized surface plasmon coupling, a fast luminescence decay produces an internal quantum efficiency enhancement^49^. The minor peak around 700 nm is the extra signal from sapphire substrate^22^.

To understand the LSPR of the small gold nanorod, a finite-element method (FEM) was chosen to simulate the optical properties of the LSPR waves of the gold nanorod. Figure 3(c) shows the calculated spatial distribution of the electric field intensity around a gold nanorod on the sapphire substrate. The optical mapping showed that high enhancement occurs in the vicinity of the nanorod, and that strongly localized optical modes propagated along the longitudinal direction at the interface of the Au and the substrate. Since the type local surface plasmonic resonances are very close to MoS_2_ monolayer, the coupling of the local plasmonic waves and the MoS_2_ emission could be much stronger. The type high-Q, long propagation length LSPR can enhance the optical emission within a small footprint, and had been applied in the ultra-compact surface plasmon nanolasers^36^,^38^,^39^. Therefore, the spectral and spatial overlapping between the gold nanorods LSPR waves and the MoS_2_ nanosheets’ emission lead to the strongly enhancement in the spontaneous emission from the single-layer MoS_2_.

**Figure 4:**
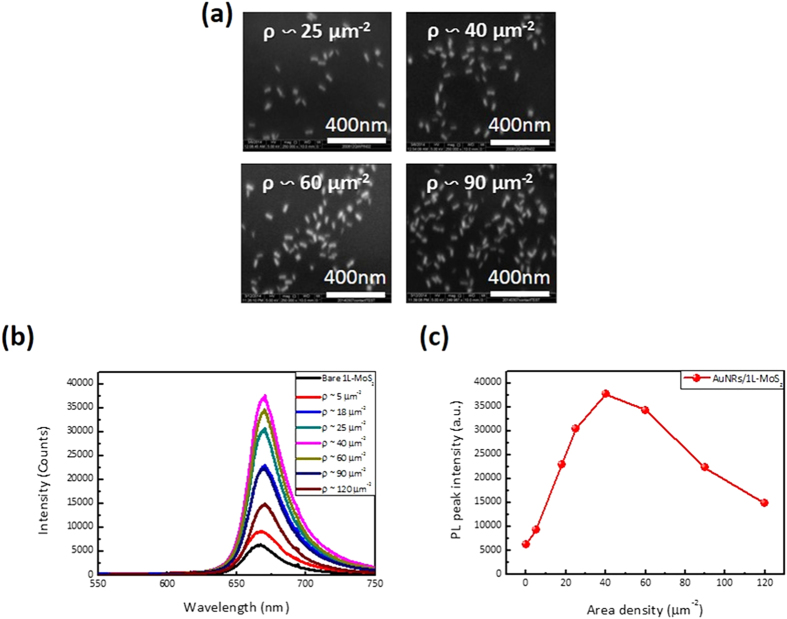
(**a**) SEM images of the gold nanords on the monolayer MoS_2_ with different densities. (**b**) The PL spectra of MoS_2_ with different gold nanorod densities. (**c**) The measured PL peak intensities of the monolayer MoS_2_ with different gold nanorod densities.

The gold nanorods in the toluene solvent with different concentrations were prepared, and then directly spin-coated on the top surface of the MoS_2_ monolayer. The aggregation of gold nanorods was not observed thanks to the nanorod decoration procedure, and the nanorod area density on the MoS_2_ nanosheet was controlled from zero to 120 μm^−2^ by varying the nanorod solution concentrations in the experiment. Figure 4(a) shows the SEM images of the nanorods on the top of the MoS_2_ with the densities of 25, 40, 60 and 90 μm^−2^. The MoS_2_ device with the gold nanorods was first characterized under the same optical pumping conditions. Figure 4(b) shows the measured PL spectra from the MoS_2_ with the different gold nanorod densities. These results indicate that the PL intensity of the MoS_2_ monolayer can be gradually enhanced by increasing the density of gold nanorods within the range of 0 to 40 μm^−2^. The redshift in the PL spectra of the MoS_2_ is attributed to the slice mismatch in wavelength between MoS_2_ emission (~670 nm) and Au nanorod resonance (~695 nm). This could be reduced if nanorod plasmonic waves matched perfectly with MoS_2_ emission in wavelength. Figure 4(c) shows that the PL-enhanced intensity as a function of the gold nanorod density on the top of the monolayer MoS_2_. However, the trend of PL enhancement gradually declined as the density of gold nanorods exceeded 40 μm^−2^. During the experiment, the Au nanorods were always placed on the top of MoS_2_. Since the thickness of the MoS_2_ monolayer is only 0.7 nm, the MoS_2_ would form the wrinkled surface if we placed the MoS_2_ on top of the Au nanorods (25 nm diameter and 57 nm length). The wrinkled MoS_2_ nanosheet would introduce the defects and the non-uniform stress, which might lead to wavelength shift and degraded intensity^26^.

The enhancement and decline of PL enhancement can be explained by external quantum efficiency of MoS_2_. For convenience, we consider the enhancement factor of emission instead of the actual count values of our experiments. First, we consider the internal quantum efficiency of our light emission material, monolayer MoS_2_. We can write down the original internal quantum efficiency, 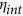, without any Au nanorods.


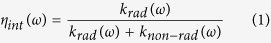



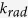
 and 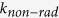 are radiative and non-radiative recombination rates, respectively. The PL signal we measured is proportional to the external quantum efficiency of the material. Therefore, we need to add another light-extraction efficiency term, 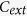, into our equation in the following way.


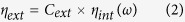


When we dispersed Au nanorods onto the MoS_2_, two factors affect to the light emission. The first one is the coupling between nanorod LSPR and the MoS_2_ emission. The second factor is the shielding effect cause by the Au nanorods on the top of nanosheet. With a single nanorod LSPR coupling, we can modify the internal quantum efficiency with nanorods, 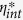, in this form.


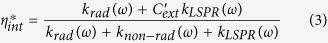



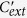
 is the probability of photon extraction from LSP’s energy, and it’s dependent on the roughness and structure of our plasmonic material. 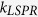 is the coupling rate between MoS_2_ and Au nanorods. This LSPR coupling results in an increase of spontaneous emission rate, and then further enhances the material’s internal quantum efficiency, 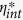
48.

However, the enhancement begins to drop as the nanorods density exceeds 40 μm^−2^. This phenomena can be explained by a simple modification of the light extraction efficiency. We consider that there are “n” nanorods inside our pumping area, “A”. And the nanrod’s length and radius are “l” and “r”, respectively. Each nanorod at the surface of MoS_2_ would somehow block the light emission. As a consequence, the modified light extraction efficiency should be written as 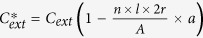. We define 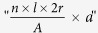 as the correction term of light extraction efficiency. And 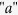 is defined as the correction factor of the enhanced area. We will explain this parameter in detail later. As we increase the density of nanorods inside our pumping area, the internal quantum efficiency, 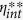, would increase and eventually saturate. So the overall effect from dispersing nanorods onto our MoS_2_ to the external quantum efficiency can be summarized as following.


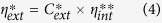



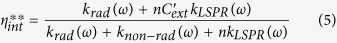



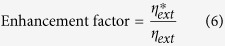


**Figure 5:**
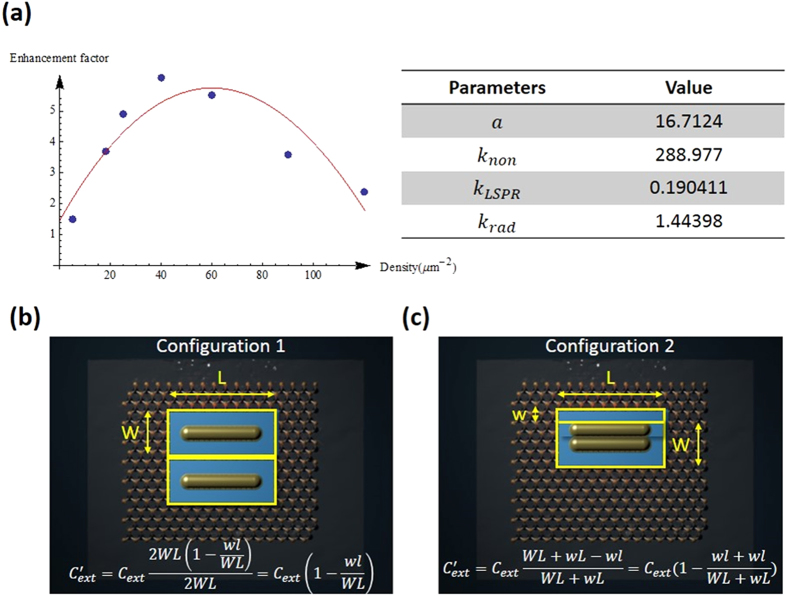
(**a**) Simulation results of enhancement factor to gold nanorod’s density. Right hand side table is the fitted parameters. (**b**) Configuration 1 is the situation that there is no overlapping of enhanced area between two gold nanorods. “W” and “L” are the enhanced area’s width and length, respectively. The equation on the bottom of the figure is the calculated external light extraction efficiency. (**c**) Configuration 2 is the extreme case that the two gold nanorods are just next to each other. “w” is the diameter of the gold nanorod.

When the density of Au nanorods is low, the 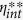 term would dominate the overall external quantum efficiency. Therefore, the PL enhancement factor is increasing as the density increases. However, as the density has reached certain level, 40 μm^−2^ in our experiment, the light extraction efficiency term starts to take over the overall external quantum efficiency, which is blocking emission from MoS_2_. Since Au metal itself will absorb the emission from MoS_2_, the same situation occurs when the density of Au nanorods is too high. We can imagine it is similar to deposit a gold film onto our device and shield all the light. And the outcome was that the enhancement is declining. Figure 5(a) is the fitting results of monolayer MoS_2_ based on previous equation. For convenience, we assume 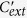, probability of photon extraction from LSP’s energy, to be 1. Although 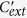 cannot be 1 in the real case, it does not influence our final results very much. The inset table of Fig. 5(a) is the fitting parameters. We can check these parameters are reasonable or not. First, we utilized the LSPR to enhance the light emission of MoS_2_. If we calculate our results for number of nanorods, 15, the internal quantum efficiency is 0.0146, which is larger than the original reported value, 4 × 10^−3^ 25.

Now we consider the correction factor of enhanced area, 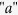. If we consider the light absorption of gold nanorods, this factor should not exceed 1. Because the thickest part of nanorod is its diameter, 25 nm. This value is still penetrable for visible light. Therefore, it should be a number between 0 and 1. From equation (1), we can easily observe that the decaying process is dominated by the light extraction efficiency, 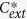. If the decaying process is not fast enough with respect to the density of nanorods, this model cannot match our experimental results. We can give a physical interpretation as following. There are two possible physical phenomenon can be the primary causes of enhancement. First one has been mentioned in the previous paragraph, which is the increment of internal quantum efficiency. Second is the emitter characteristic of gold nanorod. In this case, the nanorod served as an antenna to concentrate output light from underneath material^52^. The combined effects will be an effective enhanced area corresponding to each plasmonic structure.

Figure 5(b,c) show the schematic diagrams of enhanced area and nanorod. Blue rectangle represents the enhanced area of a nanorod. Configuration 1 shows the situation that there is no overlapping between enhanced area of two nanorods. Configuration 2 shows the extreme case that the two nanorods are next to each other. From extraction efficiency factors of both cases, we can see that it’s obvious the 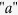 factor would exceed one in the configuration 2. If we divide the Configuration 2’s correction term, 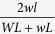, with Configuration 1’s, 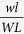, we get 
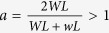
. Therefore, the overall effect of decaying process of enhancement can be attributed to the overlapping between enhanced area of nanorods and the blocking by nanorods. The effective enhanced area of single nanorod would be 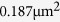. And our laser pumping area is of diameter, 2 μm. If we assume all the nanorods aligned properly and there is no enhanced area overlapping, about 16 nanorods would fill up the excitation area. And our decaying phenomena occurred at density of 40 μm^−2^, which is larger than the calculated value, indicated that the excitation area is overwhelmingly filled up with nanorods. Therefore, it is reasonable to expect a decaying in the enhancement factor. To confirm the metal object coverage effect in the MoS_2_ emission, we also prepared the MoS_2_ nanosheet coated with Au nanoparticles, which its LSPR wavelength is far away from the MoS_2_ emission wavelength (see supplemental information). In the case, the MoS_2_ emission was degraded as the nanoparticle density increased due to the shielding effect cause by the Au nanoparticles.

In our experiment, the nanorod area density was controlled between 5 to 120 μm^−2^. The Au nanorods covered less than 20% of surface area, which is relatively low coverage. But it is worth to note that, under high nanorod density situation, the extra plasmonic waves could be generated due to the plasmonic wave coupling of the nanorods. The extra plasmonic waves might cause other effects for MoS_2_ emission. We also observed the enhancement of the Raman signal of the MoS_2_ nanosheet with the Au nanorods (please refer supplemental information).

## Conclusion

In conclusion, we reported localized surface plasmon-enhanced PL of a monolayer MoS_2_ in the presence of gold nanorods that can be synthesized in large quantities. The light emission from a monolayer MoS_2_ gradually increased with the area density of gold nanorods up to 40 μm^−2^. However, the enhancement decreased when the Au nanorod density was more than 40 μm^−2^, because of the absorption of Au nanorods and overlapping of enhanced area. Therefore the nanorod density optimization is critical and necessary to obtain the maximum emission out from the 2-D TMDC nanosheet. A simple physical model was also illustrated to explain the optimum nanorod density for enhancing the emission from MoS_2_. The maximal PL enhancement factor due to the presence of these gold nanorods was approximately 6.5-fold. We note that if the main emission peak position of MoS_2_ completely overlaps the longitudinal resonant peak of gold nanorods, its intensity can be further enhanced. In addition, if the short axis of nanorod can be fine-tuned properly, we can match the short axis’ resonance to the excitation wavelength and enhance the absorption resulting in even more emission enhancement. Utilizing gold nanoparticles is a non-invasive and reliable way to enhance emission of MoS_2_. In comparison with traditional lift-off method with evaporated metal nanostrutures, the synthesized gold nanorods have better plasmonic property. And dispersing gold nanorods is lithography free. Since dispersing gold nanorods onto MoS_2_ is a physical enhancing method, this method can be integrated with other platforms such as 2-D materials-based LED and laser systems^53^,^54^. Our work gives an economic and easy way to enhance the emission of the few layers transition metal dichalcogenides.

## Methods

### CVD Growth of MoS_2_ monolayer

The growth of the monolayer MoS_2_ is based on the vapor phase reaction between MoO_3_ and S as reported in the previous studies^21^,^22^.

### Gold nanorod preparation

The Au nanorods were synthesized using the seed-mediated method in the presence of cetyltrimethylammonium bromide (CTAB) as the shape-directing agent. To render gold nanorods soluble in an organic environment, thiolated poly(ethylene glycol) was used to replace the protective CTAB bilayer on the surface of the gold nanorods. The gold nanorods were then centrifuged at 15000 rpm for 20 min, and the free CTAB-containing supernatant was discarded. Gold nanorods pellets were re-dispersed in deionized water. Centrifugation or the washing step was repeated twice. To synthesize pegylated gold nanorods, mPEG5000-SH (2 mg/mL) was added to the gold nanorod solution before it underwent sonication for 1.5 h at 50 °C. The reaction was left at room temperature overnight. The as-synthesized PEGylated gold nanorods exhibited excellent dispersibility in various organic solvents, including tetrahydrofuran and chloroform. The thickness of the PEG layer on the nanorods would be approximately 5 nm, which is the distance between nanorods and MoS_2_ monolayer. Therefore the coupling between MoS_2_ emission and NR plasmonic waves is very strong, which leads to the strong enhancement in PL.

### Characterization

Raman spectra and photoluminescence measurement were performed in a Jobin-Yvon Horiba HR800 micro-Raman spectroscope at room temperature. All spectroscopy measurements were obtained with a confocal microscopy setup in a back-scattering geometry using a CW DPSS laser, at a wavelength of 488 nm (the spot size of the laser was approximately 2 μm in diameter). To prevent sample overheating, an excitation power of 20 μW below the microscope objective lens was normally injected on the MoS_2_.

## Additional Information

**How to cite this article**: Lee, K. C. J. *et al.* Plasmonic Gold Nanorods Coverage Influence on Enhancement of the Photoluminescence of Two-Dimensional MoS_2_ Monolayer. *Sci. Rep.* **5**, 16374; doi: 10.1038/srep16374 (2015).

## Supplementary Material

Supplementary Information

## Figures and Tables

**Figure 1:**
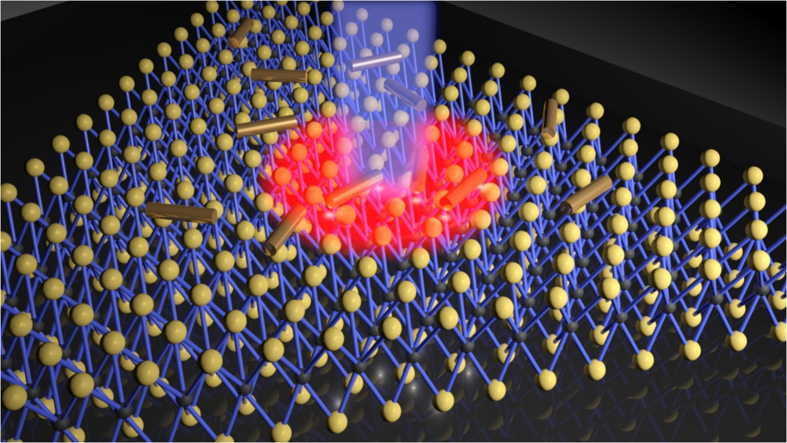
Schematic diagram showing optical enhancement from the gold nanorods with strongly localized surface plasmon waves around the nanorod’s ends and MoS_2_ interface (White spots). The middle blue cylinder is our pumping 488 nm laser. And the red region is the emission area of MoS_2_.
