# Supplementary material for: Plasmonic Gold Nanorods Coverage Influence on Enhancement of the Photoluminescence of Two-Dimensional MoS2 Monolayer
Source: Sci Rep. 2015 Nov 17;5:16374. doi: 10.1038/srep16374 (PMC4647184; doi:10.1038/srep16374)
Supplement: Supplementary Information [file srep16374-s1.pdf]

SUPPLEMENTAL INFORMATION

Plasmonic Gold Nanorods Coverage Influence on Enhancement of the Photoluminescence of Two-Dimensional Two-Dimensional MoS_2 Monolayer

Kevin C. J. Lee,[1] Yi-Huan Chen,[1] Hsiang-Yu Lin,[1,2] Chia-Chin Cheng,[3] Pei-Ying Chen,[5] Ting-Yi Wu,[1,2] Min-Hsiung Shih,[1,2,6,*], Kung-Hwa Wei,[3] Lain-Jong Li,[4] and Chien-Wen Chang[5]

[1]*Research Center of Applied Sciences (RCAS), Academia Sinica, Taipei, 11529, Taiwan*

[2]*Department of Photonics, National Chiao Tung University (NCTU), Hsinchu, 30010, Taiwan*

[3]*Department of Materials Science & Engineering National Chiao Tung University Hsinchu, 30010, Taiwan*

[4]*Physical Science and Engineering Division, King Abdullah University of Science and Technology (KAUST), Thuwal, 23955-6900, Kingdom of Saudi Arabia*

[5]*Department of Biomedical Engineering and Environmental Sciences, National Tsing Hua University (NTHU), Hsinchu, 30013, Taiwan*

[6]*Department of Photonics, National Sun Yat-sen University, Kaohsiung, 804, Taiwan*

MoS_2 Nanosheet Coated with Gold Nanaoparticles

To confirm the PL enhancement caused by the coupling between Au LSPR waves and the MoS_2 emission, we also prepare a reference MoS_2 nanosheet coated with the Au nanoparticles, which its LSPR wavelength deviates from the MoS_2 emission spectrum. The spherical Au nanoparticles with a diameter of 15 nm were placed on the top of the reference devices with the same coating procedure. The black curve in Figure S1(a) represents its absorption spectrum, and the LSPR peak was at a wavelength of approximately 556 nm, which is far from the emission peak of MoS_2 (the red curve in Figure S1(a)). The inset in Figure S1(a) is an SEM image of the Au nanoparticles on the MoS_2 surface. Figure S1(b) shows the measured PL spectra from the MoS_2 nanosheet with different Au nanoparticle densities, ranging from 0 to 335 μm^{-2} , and Figure S1(c) shows the PL peak intensity of the MoS_2 nanosheet with different nanoparticle densities. Because of the spectral mismatch of Au nanoparticle LSPR and MoS2 emission, the PL peak (~670 nm) intensity of the MoS_2 with gold nanoparticles was degraded as the Au nanoparticle coverage increased. The behavior serves an evidence of the shielding effect because the excessive metallic objects absorbed MoS_2 emission.

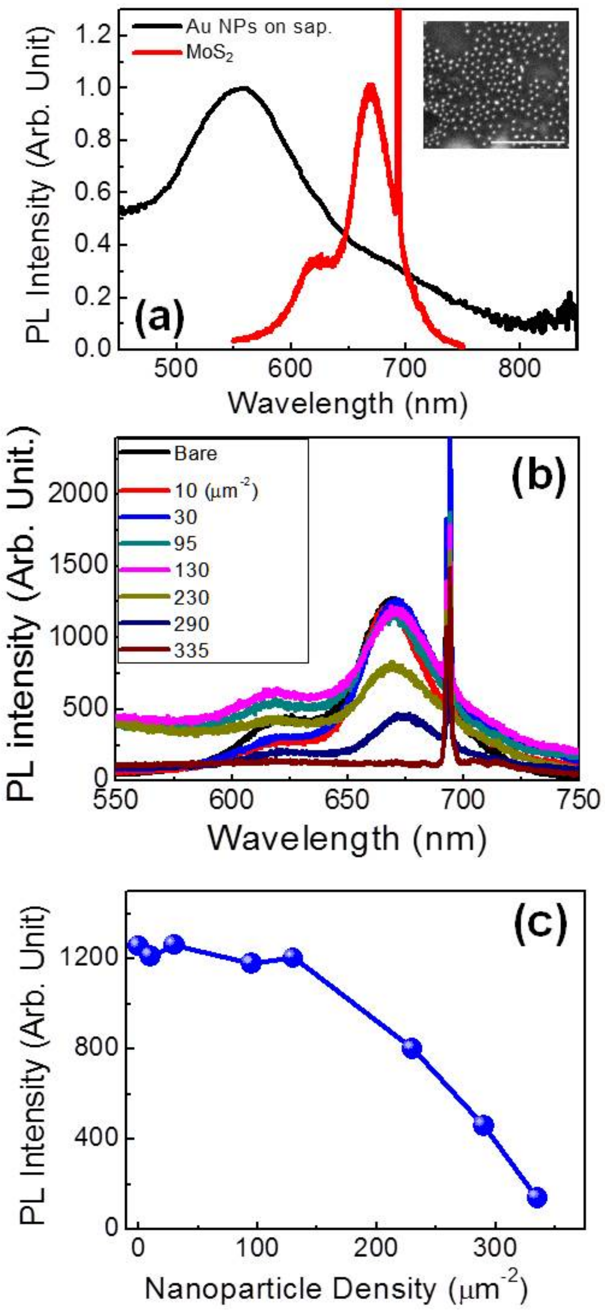

Figure S1 (a) The extinction spectra of the gold nanoparticles and PL spectra of the MoS_2 nanosheet. (b) PL spectra of gold nanoparticle-coated MoS_2 nanosheet with different area densities. The inset presents an SEM image of gold particles on the surface of the MoS_2. (c) PL intensity of the MoS_2 with the different densities of gold nanoparticles.

Raman Signal Enhancement of the MoS2 Nanosheet with Gold Nanorods

The Raman signals of the MoS_2 under the different Au nanorod coverages were also characterized with the 488 nm wavelength pumping laser. Figure S2 shows the Raman spectra of MoS_2 with different nanorod densities. Two Raman peaks of the MoS_2 nanosheet were observed at 385 cm^{-1} and 405 cm^{-1}. The intensity of Raman signals increased as the Au nanorod density increased. This behavior was attributed to the coupling between the pumping laser signal and the LSPR (~ 500 nm wavelength in the PL (black) spectrum of Figure 3(b)) along short-axis of nanorod. This surface-enhanced Raman spectroscopy (SERS) enhancement was not very high, compare to the reported values (10^6 ~ 10^{14}) , due to the weaker LSPR waves along the short-axis of Au nanorod.

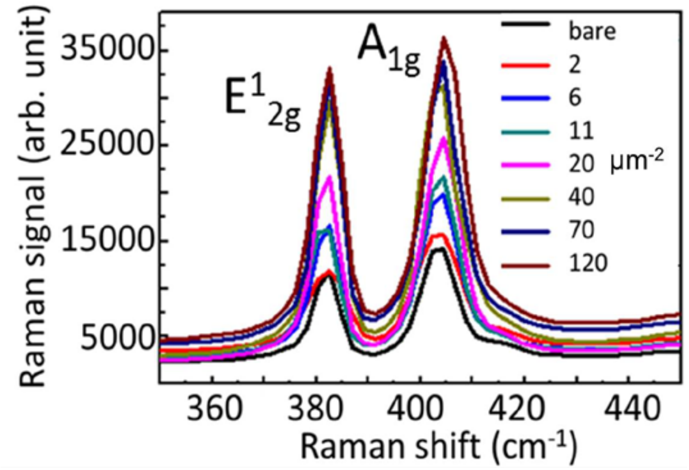

Figure S2 Raman spectra of the MoS_2 nanosheet with the different densities of Au nanorods.
